# Pneumonectomy for broncho-pulmonary carcinoids: a single centre analysis of surgical approaches and patient outcomes

**DOI:** 10.3389/fonc.2024.1383352

**Published:** 2024-07-24

**Authors:** Cristina Diotti, Luca Bertolaccini, Lara Girelli, Clarissa Uslenghi, Stefano Maria Donghi, Juliana Guarize, Francesca Spada, Nicola Fazio, Lorenzo Spaggiari

**Affiliations:** ^1^ Department of Thoracic Surgery, IEO, European Institute of Oncology IRCCS, Milan, Italy; ^2^ Division of Interventional Pulmonology, IEO, European Institute of Oncology IRCCS, Milan, Italy; ^3^ Division of Neuroendocrine and Digestive Oncology, IEO, European Institute of Oncology IRCCS, Milan, Italy; ^4^ Department of Oncology and Hemato-Oncology, University of Milan, Milan, Italy

**Keywords:** pneumonectomy, neuroendocrine tumors, lung cancer, perioperative management, outcomes, survival analysis

## Abstract

**Background:**

Pneumonectomy is a radical surgical procedure associated with significant morbidity and mortality. Its application in the context of pulmonary neuroendocrine tumours, including carcinoid tumours, requires meticulous preoperative planning and intraoperative precision. This study aims to assess the safety and efficacy of pneumonectomy in the management of these rare and challenging neoplasms.

**Methods:**

A retrospective analysis of patients who underwent pneumonectomy for pulmonary carcinoid tumours at our institution over a specified period was conducted. Data regarding patient demographics, tumour characteristics, surgical techniques, intraoperative complications, perioperative management, and long-term outcomes were collected and analysed.

**Results:**

Between March 2001 and October 2022, 21 patients (7 male, 14 female) with carcinoid tumours underwent pneumonectomy on a total of 459 surgical operations for carcinoid. Preoperative bronchoscopic procedures were conducted in 90.4% of cases, leading to histological diagnoses for most. The median hospital stay was eight days, with no reported perioperative deaths. Median follow-up after surgery was 73 months, with a five-year overall survival of 65.4 months. Recurrences occurred in 28.6% of cases, primarily in atypical carcinoids.

**Conclusion:**

Despite the rarity of bronchial carcinoids, pneumonectomy is effective for low-grade malignancies, demonstrating positive short—and long-term outcomes. Radical lymph node dissection is fundamental in pathological staging and overall survival.

## Introduction

According to the 2015 World Health Organization classification of neuroendocrine tumours (NETs), bronchopulmonary carcinoids are rare, well-differentiated bronchial NETs, accounting for 1 – 2% of all lung cancers. They have relatively indolent biological features but potentially aggressive behaviour, with loco-regional and extrathoracic metastatic spreading in almost 10% of cases (1, 2).

Morpho-pathological characteristics have classified broncho-pulmonary carcinoids as typical carcinoids (<2 mitoses/2 mm^2^ of viable tumour and lacking necrosis) and atypical carcinoids (2 – 10 mitoses/2 mm2 of viable tumour, presence of necrosis and/or architectural disruption). Typical carcinoids <5 mm are defined as carcinoid tumourlets. This condition can lead to idiopathic neuroendocrine cell hyperplasia (DIPNECH), a precursor for carcinoid tumour development (3). Even if surgery is the treatment of choice for non-metastatic disease (4, 5), the best surgical approach for the treatment of bronchial carcinoids is not yet defined by international guidelines and substantially depends on the location and the extent of the tumour itself (6). At present, anatomical resections with radical lymphadenectomy are considered necessary to ensure adequate radicality. In case of centrally-located lesions or endobronchial extension not allowing parenchyma-sparing bronchoplastic procedures, an appropriate surgical approach may require a pneumonectomy, even if its role in low-grade malignancies is still debated (7). Endobronchial treatment is an alternative for symptom relief for patients for whom surgery is contraindicated or for patients who require a delay in surgery (8).

This study aims to assess the safety and oncological outcomes of pneumonectomy in the management of broncho-pulmonary carcinoids.

## Materials and methods

The Institutional Review Board, informed of the database extraction, did not require approval because of the study’s retrospective nature. The authors had no information to identify individual participants during or after data collection. This manuscript was written according to the Strengthening the Reporting of Cohort Studies in Surgery (STROCSS) Statement (9). The STROCSS checklist is available as **Supplementary File 1**.

We performed a single institution experience retrospective analysis reviewing clinical records of patients who underwent pneumonectomy for bronchial carcinoid over more than 20 years. Between 1 March 2001 and 31 October 2022, on a total of 459 surgical operations for broncho-pulmonary carcinoids, 21 (4.6%) pneumonectomies with a definitive histological diagnosis of carcinoid were performed. Clinical evaluation has varied over the years based on the latest guidelines’ updates (7). In recent years, diagnosis and staging included whole-body Computed Tomography (CT) scan and positron emission tomography (PET)/CT with Gallium-68 (^68^Ga)-labelled somatostatin analogues (SSAs). A preoperative bronchoscopic examination was routinely performed to evaluate bronchial invasion, obtain endoscopic biopsies, and sample hilar and mediastinal lymph nodes using EndoBronchial UltraSound-guided TransBronchial Needle Aspiration (EBUS-TBNA).

Furthermore, pulmonary function tests were performed to plan a pneumonectomy, including circulation of single breath diffusing capacity for carbon monoxide, lung perfusion scan, and cardiopulmonary exercise testing. All patients were discussed with a skilled neuroendocrine multidisciplinary team. Written informed consent was obtained at hospital admission to use patients’ health data for therapeutic purposes and clinical trials.

Pneumonectomy was the only surgical approach to obtain radicality; lung-sparing surgery, even with bronchoplasty, was impossible (e.g. centrally located endobronchial lesions invading the main bronchus or hilar lesions involving vascular structures). Pneumonectomy was standardly performed via lateral muscle-sparing thoracotomy and was always accompanied by a radical Hilo-mediastinal lymphadenectomy, as recommended by NCCN Clinical Practice Guidelines (4). R0 radicality was ensured by intraoperative analysis of the bronchial margin at the frozen section.

The patient demographics and clinicopathological characteristics included age, sex, smoke history, previous malignancy, side, surgical approach, tumour size, number of lymph nodes dissected and pathologic nodal stations, pathological stage, and neoadjuvant and adjuvant treatments (**Table 1**). Histopathological features included Ki-67, mitosis/10 HPF, necrosis, and grading. Data collection was completed with patient perioperative outcomes regarding ICU stay, discharge after surgery, postoperative complications, and long-term outcomes, particularly overall survival (OS) and disease-free survival (DFS). Each patient underwent regular follow-up with a periodic CT scan. Data related to the recurrence site and treatment were also collected in case of recurrence.

**Table 1:** Patients’ characteristics.

	Typical Carcinoid (No. = 7)	Atypical Carcinoid (No. = 14)	*p-value*
**Age, median (range)**	45 (25 – 70)	52 (29 – 74)	*0.93*
**Sex** **Male** **Female**	3 4	4 10	*0.51*
**Side** **Right** **Left**	5 2	4 10	*0.061*
**Ki-67%, median (range)**	2 (1 – 7)	8 (1 – 35)	*0.49*
**Tumour size (mm), median (range)** **pT** **pT1** **pT2** **pT3** **pT4** **pN** **pN0** **pN1** **pN2**	58 (15 – 103) 1 1 2 3 3 3 1	41 (17 – 58) 1 8 3 2 5 2 7	*0.18*
**Number of harvested N1 lymph nodes, median (range)**	15 (9 – 20)	18 (6 – 28)	*0.46*
**Number of harvested N2 lymph nodes, median (range)**	8 (3 – 12)	7 (2 – 14)	*0.81*
**Neoadjuvant treatments** **None** **Chemotherapy** **Chemo-Radiotherapy**	7 0 0	10 2 2	*0.29*

### Statistical analysis

Quantitative variables were expressed as median (range), whereas nominal variables were defined binarily as the presence or absence of the event. Kruskal – Wallis Rank test was used for continuous variables, and the Fisher Exact test was used for categorical variables. Median OS and DFS were estimated using the Kaplan – Meier method. The log-rank test compared the differences in survival rates. A p-value of less than 0.05 was considered significant for all the statistical analyses. The *EZR*, *irr*, and *rcmdr* packages were used in RStudio (R version 4.2.1, Funny-Looking Kid) for statistical analysis (Team R. RStudio: Integrated Development Environment for R. Boston, MA: RStudio, Inc.; 2021. Team RC. R: A Language and Environment for Statistical Computing. Vienna, Austria: R Foundation for Statistical Computing; 2021.).

## Results

Between March 2001 and October 2022, we included 21 patients (7 male, 14 female) with a median age of 50 years (range: 25 – 74 years). Demographics are described in **Table 1**. Almost all (90,4%) patients underwent a preoperative bronchoscopic procedure to evaluate bronchial involvement and acquire a tissue sample. In two patients, we did not collect a histological diagnosis. In two cases, a preoperative diagnosis of adenocarcinoma was then disproved by a definitive histological diagnosis of carcinoid. The tumour was located in the right lung in 9 (42.9%) patients and the left in 12 (57.1%) patients. The median tumour size for Typical Carcinoid was 58 mm (range: 15 – 103 mm). The median tumour size in the Atypical Carcinoid group was 41 mm (range: 17 – 58 mm). The median Ki-67 for Typical Carcinoids was 2% (range: 1 – 7%), whereas in the Atypical Carcinoids group was 8% mm (range: 1 – 35%) and did not show statistically significant differences (p = 0.49). The distribution of tumours across pT stages and lymph node involvement (pN) did not show statistically significant differences between Typical Carcinoid and Atypical Carcinoid groups (p = 0.46 and p = 0.81, respectively). The distribution of patients based on neoadjuvant treatments did not exhibit a significant difference between the Typical Carcinoid and Atypical Carcinoid groups (p = 0.29). All patients were discussed in a skilled multidisciplinary neuroendocrine tumour board. All lesions were centrally located with a maximum diameter ranging from 15 to 103 mm; therefore, lung-sparing surgery with bronchoplasty was considered technically not feasible or inadequate to obtain surgical radicality. Four patients underwent neoadjuvant treatments: two of them were treated with chemotherapy and two with combined chemo and radiotherapy before surgery. Two patients were misdiagnosed before surgery (one SCLC, considered for surgery as very limited disease, and one adenocarcinoma); the other two cases were diagnosed as well-differentiated neuroendocrine tumours, and a patient-tailored preoperative therapy was proposed after multidisciplinary board; one case of single bone metastasis and the other of bulky N2 disease were treated before surgery. Definitive histologies collected typical carcinoids in 7 (33.3%) and atypical carcinoids in 14 (66.7%) patients. A median of 17 N1 station and 7 N2 station lymph nodes were harvested; 8 cases of pN0, 5 cases of pN1, and 8 cases of pN2 disease were recorded. One patient with pN2 disease was treated with adjuvant therapy.

The median hospital stay after pneumonectomy was eight days (range: 5 – 24 days). No 30-day perioperative deaths were reported. Four (19.0%) postoperative surgical significant complications were reported: two (9.5%) cases of haemothorax requiring reoperation, one (4.8%) bronchopleural fistula (occurred a month after surgery), and one (4.8%) oesophageal-pleural fistula (occurred six months after surgery). An empyema was associated with both bronco-pleural and pleural fistulas. The bronchopleural fistula was closed by a direct suture reinforced with pledges and covered with an intercostal muscle flap; the oesophagus-pleural fistula was repaired using a pectoralis major muscle flap after oesophageal stent endoscopic placement.

**Figure 1:**
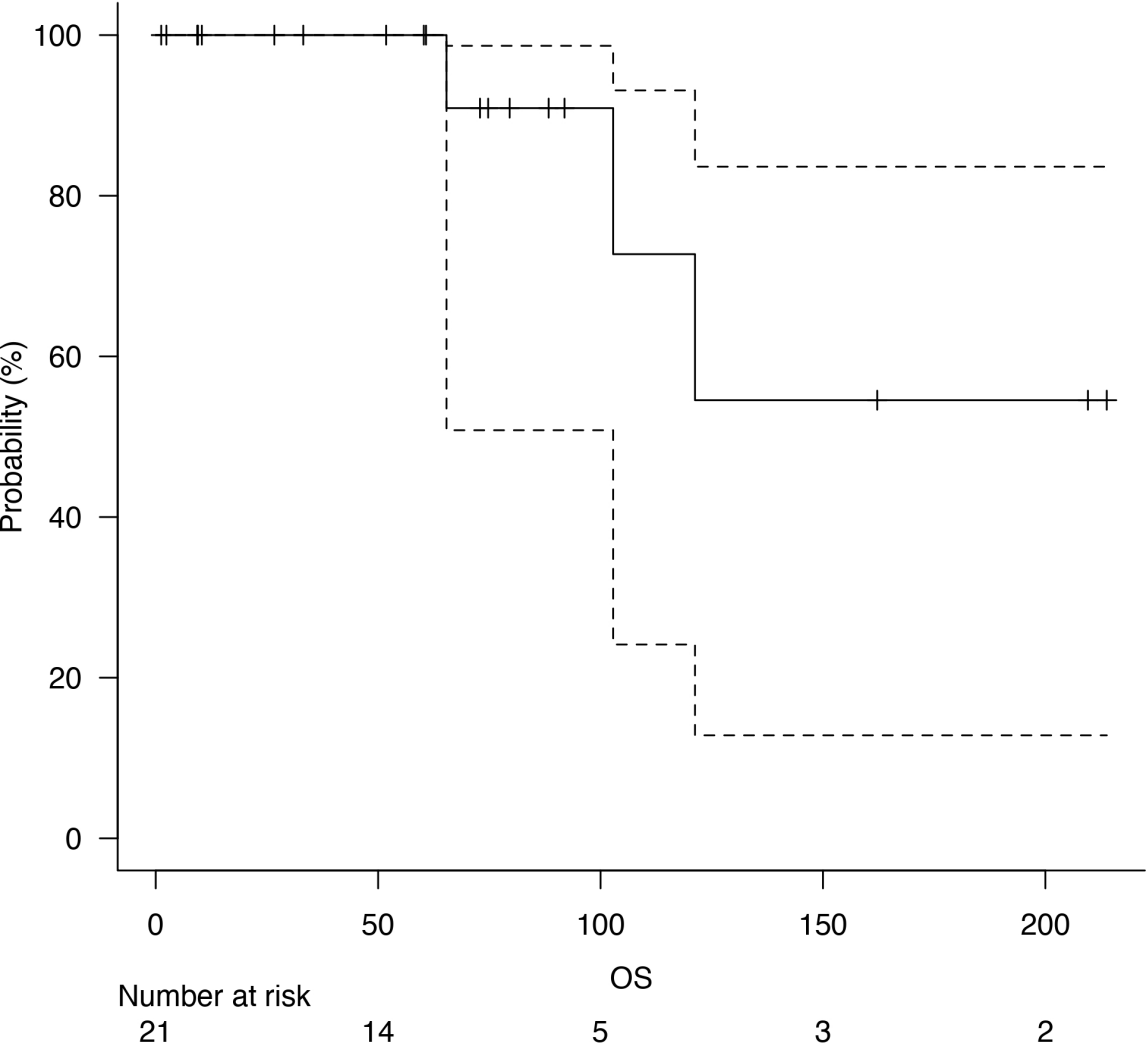
Overall survival (OS).

**Figure 2:**
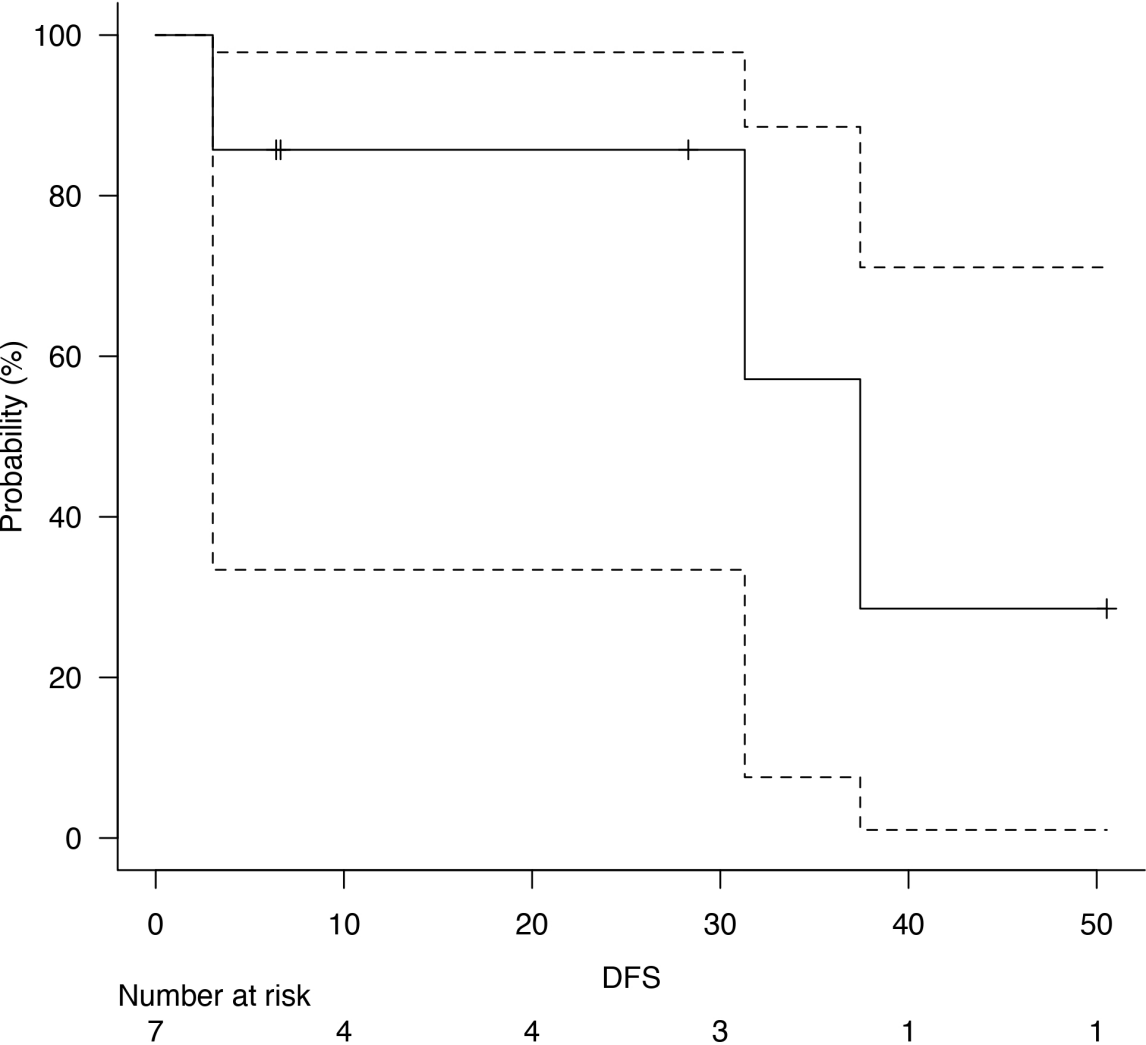
Disease-free survival (DFS).

**Figure 3:**
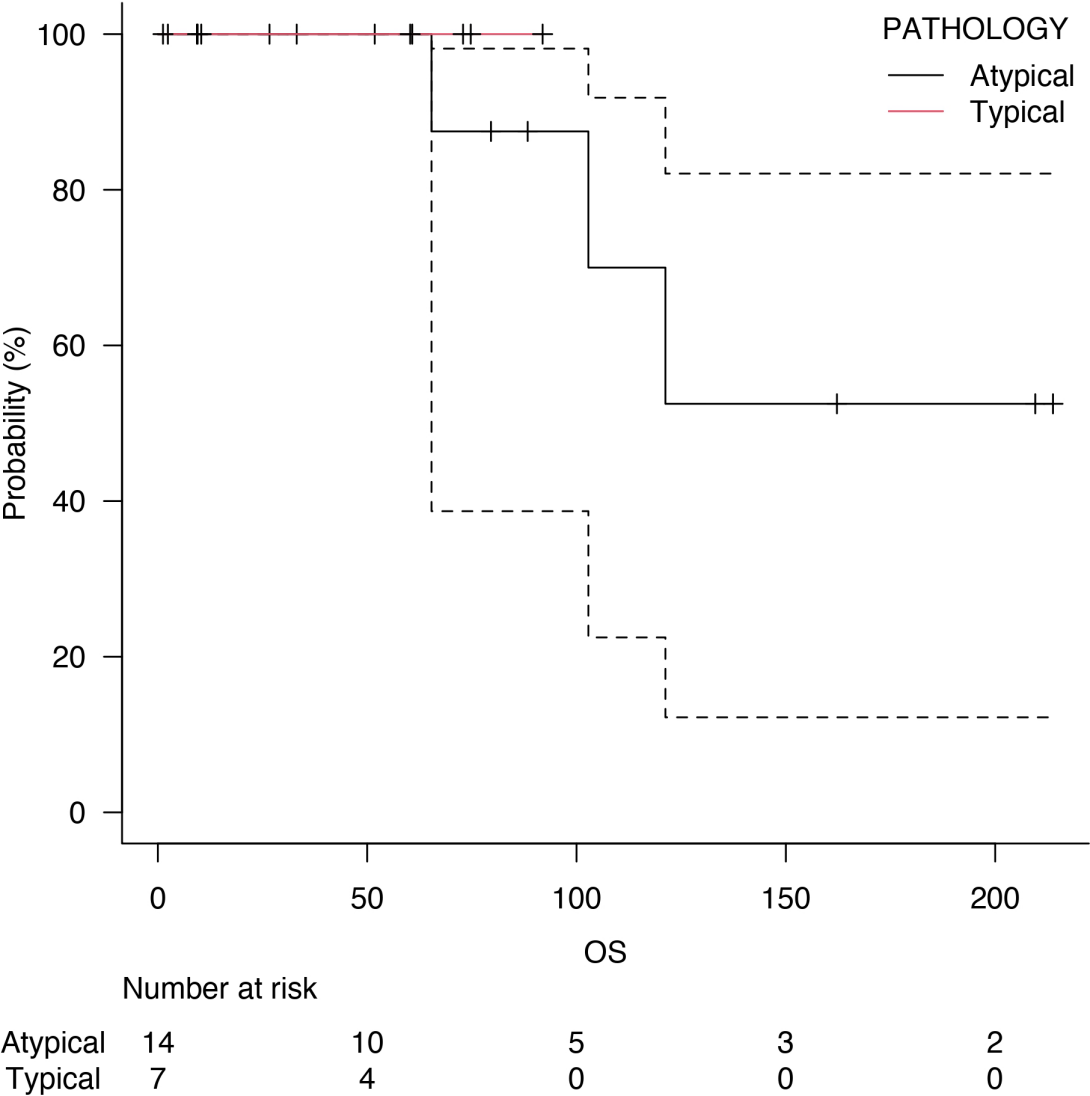
Overall survival (OS) of typical carcinoids (red line) and atypical carcinoids (black line).

**Figure 4:**
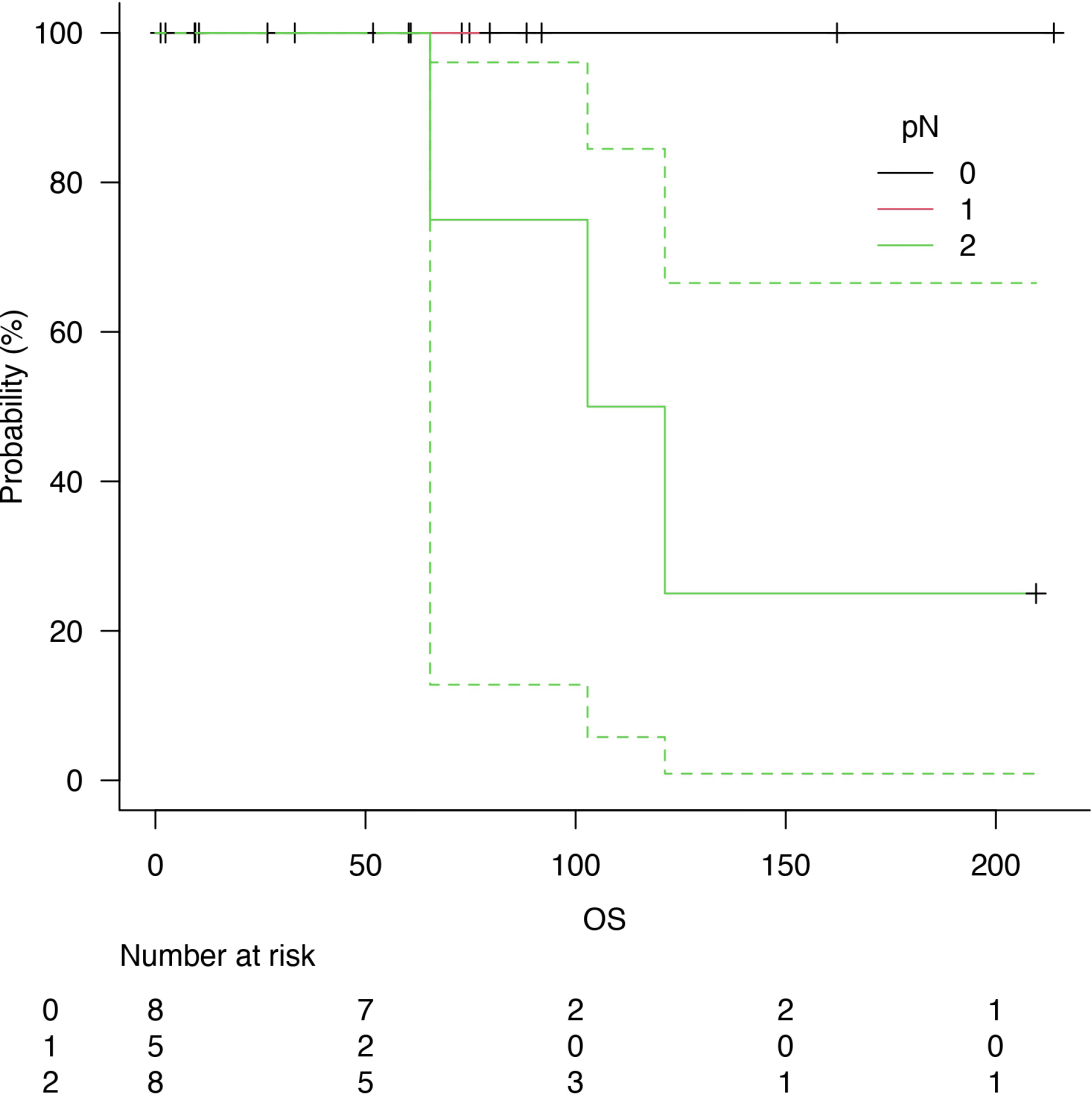
Overall survival (OS) related to nodal status: N0 (black line), N1 (red line) and N2 (green line).

Follow-up was at least six months for every patient, with a median follow-up of 73 months after surgery. Five years of median OS after pneumonectomy was 65.4 months (**Figure 1**). The median DFS was 23 months, with a median survival after diagnosis of recurrence of 71 months (**Figure 2**). Disease recurrences were recorded in 6 (28.6%) patients; in 3 (14.3%) patients, mediastinal nodal recurrence was evidenced, and in 3 (14.3%) patients showed distant metastases (liver and bone). Two patients had both intrathoracic and distant metastases. All the recurrences were related to the atypical carcinoid subtype. On the contrary, there was no significant correlation between the ranges of Ki-67 and the recurrences (p = 0.67). In the case of recurrent disease, most patients were treated with somatostatin analogues (e.g., Lanreotide); single hepatic and bone lesions were treated with local therapies (radiotherapy or thermal ablation with radiofrequency). The survival outcomes between pathological subtype groups showed a significant trend (log-rank trend test, p = 0.048) in survival differences based on the typical pathology of carcinoids (**Figure 3**). The compared survival outcomes based on the pN stage suggest a significant difference (log-rank trend test, p = 0.039) in survival among the pN stages (**Figure 4**).

## Discussion

Surgery is considered the mainstay of treatment in case of stage I – III bronchial carcinoids: anatomic pulmonary resections (e.g., segmentectomy, lobectomy, bilobectomy) or bronchoplastic procedures (e.g., sleeve resections) together with radical lymph node dissection are recommended in localised disease (Grade IV, B recommendation) (5). Hilar bronchial or endobronchial carcinoids causing obstructive pneumonia or not considered resectable with a parenchyma-sparing bronchoplastic procedure or bronchial sleeve resection may require a pneumonectomy. In these patients, a careful risk-benefit analysis should consider several factors, both the tumour and the patient: surgical extended resections for bronchial carcinoids are a matter of debate, especially in the present era of minimally invasive lung-sparing surgery. Surgical resection should include systematic hilar and mediastinal homolateral nodal dissection: nodal metastases have an incidence of up to 27% for typical and 47% for atypical carcinoids (5, 10). The manuscript does not compare the safety of pneumonectomy and carcinoids with NSCLC. Nevertheless, to our knowledge, this is the first report of a series of pneumonectomies for carcinoids.

Preoperative biological features of the tumour (typical vs atypical subtype, Ki-67, presence of necrosis) are also relevant. However, preoperative diagnosis in the case of carcinoid tumours may be demanding for pathologists because of the difficulty of differentiating tumour subtypes from biopsy or fine-needle aspiration samples. In 2021, the WHO Thoracic Tumors Classification discouraged the gradation of lung carcinoids in biopsies (11). Reuling et al. suggest that if carcinoid differentiation is clinically relevant, a cumulative biopsy size of at least four mm^2^ should be sampled (12). Most of our preoperative diagnoses were obtained with endoscopic biopsies. Since 2011, EBUS-TBNA has been routinely performed at our institution to assess nodal involvement, particularly in centrally-located tumours. In our case series, two cases of carcinoid were misinterpreted as carcinomas before surgical resection.

Moreover, a recent analysis of the US National Cancer Database conducted on more than 6000 carcinoid tumours treated surgically showed nodal upstage in 16% of atypical and 7% of typical carcinoid patients (13). A multicentric, retrospective study investigating which factors may predict nodal upstaging in lung carcinoids evidenced that atypical histology, tumour dimension, and central location are associated with a high risk for occult Hilo-mediastinal metastases (14). These data underline the pivotal role of radical node dissection even in carcinoid tumours: the presence of lymph node involvement may affect prognosis, being an independent predictor of local recurrence and worse survival, with a significantly worse prognosis in nodal positive atypical carcinoids (15–19).

Considering the patient’s characteristics, preoperative functional respiratory and cardiac tests are mandatory to evaluate surgical feasibility and predict possible postoperative complications; however, the median age of patients affected by carcinoid tumours is lower than that of patients with other lung tumours (55 years for carcinoid tumours vs. 70 years for NSCLC (5, 18)). Preoperative functional tests are often permissive also for major anatomical lung resections such as pneumonectomy.

Endobronchial treatment (EBT) may be an alternative to surgery only in very selected cases: it can be an option to obtain bronchial disimpaction and symptom relief in obstructive pneumonia, but only in rare cases is it considered a radical treatment. Van der Heijden et al. proposed modifying current guidelines of the National Comprehensive Cancer Network (NCCN) and the European Neuroendocrine Tumour Society (ENETS) – both recommending surgical resection as the treatment of choice for most localised carcinoid tumours – suggesting EBT as the first-line treatment for intraluminal typical carcinoids < 20 mm regardless of the histological grade (20), but clinical shreds of evidence were not robust enough to support such modification, reporting only 2-years overall survival and considering that only 5-10% of carcinoids are polyp-like without invasion of the bronchial wall (20). In our case series, one patient previously treated endoscopically had an endobronchial recurrence of atypical carcinoid diagnosed with bronchial biopsies: local recurrence was at the origin of the intermediate bronchus, parenchyma-sparing bronchoplastic procedure was not feasible and a right pneumonectomy was performed. One of 25 N1 resected lymph nodes resulted in a positive, showing the importance of radical node dissection and the inadequacy of endoscopic treatments, even in the case of low-grade malignancies. Nevertheless, endobronchial definitive treatment is discouraged and performed only for debulking in patients who cannot tolerate major surgery or to reduce symptoms caused by bronchial obstruction (21).

Preoperative bronchoscopic tumour ablation appears beneficial for bronchopulmonary carcinoid tumours, though long-term data is scarce. In a cohort study of 208 patients, the Procedure of Endobronchial Preparation for Parenchyma-sparing Surgery was investigated. Among centrally located carcinoids, 77 patients underwent preoperative recanalisation, leading to a higher rate of subsequent parenchyma-sparing surgeries. Ten-year survival rates were 89% for typical and 68% for atypical carcinoids. PEPPS slightly improved long-term survival without impacting metastasis or recurrence rates. This suggests that preoperative bronchoscopic treatment facilitates parenchyma-sparing surgeries without adverse effects on outcomes (22). Another study aimed to evaluate the long-term outcomes of initial bronchoscopic treatment in patients with intraluminal bronchial carcinoids due to their classification as low-grade malignancies. Initial bronchoscopic treatment improved presurgical conditions, obtained tissue samples for histologic classification and enabled less extensive parenchymal resection. High-resolution computed tomography and bronchoscopy differentiated intraluminal versus extraluminal tumour growth, with surgery following for atypical carcinoids, residue, or recurrence. Among 72 patients treated, with a median age of 47 and a median follow-up of 65 months, 79% had typical carcinoids. Initial bronchoscopic treatment achieved complete tumour eradication in 46% of cases, with 51% requiring surgery, primarily for atypical carcinoids or late-detected recurrences. Only one death was tumour-related. The study suggests initial bronchoscopic treatment as a potentially less invasive alternative to immediate surgical resection for intraluminal bronchial carcinoids, with excellent long-term outcomes and no adverse impact on surgical treatment outcomes (23).

Endoscopic debulking does not help avoid pneumonectomy because even if the endobronchial component can be effectively removed, we must consider an eventual bronchial wall/submucosal invasion. In addition, endoscopic treatment does not reduce the surgery volume and should be used only to relieve symptoms. In our series, only one patient was treated endoscopically before surgery, and a pneumonectomy was performed after an early local recurrence (24).

All these patients were discussed before surgery in a specific neuroendocrine tumours multidisciplinary team. In our institution, we support and realise parenchyma-sparing surgery for carcinoids. The number of pneumonectomies is meagre compared to the volume of surgery for non-small cell lung cancers in our Surgical Department. In the patients included in this series, sleeve resections were not technically feasible, and pneumonectomies were the only pathway to follow since oncological radicality is our common goal, particularly for young patients. A definitive histological differentiation between typical and atypical was possible only after surgery in a patient. On the other hand, we strongly discourage endoscopic treatment whenever surgery is feasible (even in the case of a typical carcinoid).

### Limitations

The limits of this study were the small sample size, the single-centre setting, the heterogeneity of the sample, and the lack of a control group, which decreased the power and significance. Secondly, the database has no information regarding the postoperative quality of life. We should have limited these weaknesses as a high-volume referral centre for neuroendocrine neoplasms.

## Conclusion

The surgical indication for pneumonectomy for bronchial carcinoids remains uncommon. In the case of centrally located tumours or endobronchial involvement, pneumonectomy should also be considered the treatment of choice in low-grade malignancies such as bronchial carcinoids with good short—and long-term postoperative outcomes. A fundamental role of radical lymph node dissection in the pathological staging of the disease and overall survival was also demonstrated. Nevertheless, bronchial carcinoid remains a rare disease, and when possible, a parenchyma-sparing bronchoplastic procedure should be selected for pneumonectomy.

## Data availability statement

The original contributions presented in the study are included in the article/**Supplementary Material**. Further inquiries can be directed to the corresponding author.

## Ethics statement

Ethical approval was not required for the study involving humans in accordance with the local legislation and institutional requirements. Written informed consent to participate in this study was not required from the participants or the participants’ legal guardians/next of kin in accordance with the national legislation and the institutional requirements.

## Author contributions

CD: Conceptualization, Data curation, Formal analysis, Funding acquisition, Investigation, Methodology, Project administration, Resources, Software, Supervision, Validation, Visualization, Writing – original draft, Writing – review & editing. LB: Conceptualization, Data curation, Formal analysis, Funding acquisition, Investigation, Methodology, Project administration, Resources, Software, Supervision, Validation, Visualization, Writing – original draft, Writing – review & editing. LG: Writing – original draft, Writing – review & editing. CU: Writing – original draft, Writing – review & editing. SD: Writing – original draft, Writing – review & editing. JG: Methodology, Supervision, Writing – original draft, Writing – review & editing. FS: Writing – original draft, Writing – review & editing. NF: Writing – original draft, Writing – review & editing. LS: Methodology, Supervision, Writing – original draft, Writing – review & editing.
